# Polytetrafluoroethylene and Aluminum Powder as an Alternative to Copper in Car Brakes Composite Friction Materials

**DOI:** 10.3390/ma18030589

**Published:** 2025-01-28

**Authors:** Andrzej Borawski, Dariusz Szpica, Grzegorz Mieczkowski

**Affiliations:** Faculty of Mechanical Engineering, Bialystok University of Technology, 45C Wiejska Str., 15-351 Bialystok, Poland; a.borawski@pb.edu.pl (A.B.); g.mieczkowski@pb.edu.pl (G.M.)

**Keywords:** brakes, friction, copper free, composite, coefficient of friction

## Abstract

Brakes are one of the most important systems of every vehicle. They have an undoubted impact on safety. Their effects produce wear products, which in the case of conventional composition of friction materials also means the content of copper in compounds emitted into the atmosphere. Its harmful effect makes it necessary to look for an alternative that will replace its excellent lubricating and thermal properties. This article presents prototype materials in which attempts were made to replace copper with powdered aluminum and polytetrafluoroethylene. Four types of samples were prepared—one group with a conventional composition, and three groups with an alternative composition, in different proportions. Using the previously developed methodology, friction tests were performed. As a result, the values of friction coefficients and abrasive wear rate were determined. The results show that the proposed material is characterized by lower values of the coefficient of friction and a higher value of the abrasive wear rate coefficient.

## 1. Introduction

The automotive industry of today’s reality is very highly developed. Life without road transport, both passenger and freight, would be much more difficult, and maintaining the current standard of living would be virtually impossible [1]. For this reason, the number of vehicles on our roads is constantly growing. It is estimated that by 2030 it will exceed 2 trillion [2]. Unfortunately, such a large number of vehicles translates into the amount of pollutants produced [3,4]. These include both pollution with gases resulting from fuel combustion [5,6] and the emission of wear particles from friction materials such as clutches, tires, and above all, brakes [7,8]. Since at present pollutant emissions from brakes pose a greater threat than in the case of exhaust fumes, great emphasis is placed on the ecological aspects of composite friction materials dedicated to vehicle braking systems [9,10].

Braking systems used in vehicles are based on the phenomenon of friction. The kinetic energy of the movement is converted into thermal energy, which makes the brakes a kind of engine—they change one type of energy into another. The amount of thermal energy obtained in the braking process depends primarily on the total mass of the vehicle, as well as on the initial speed [11,12,13,14,15,16].

The most common structural solutions currently used are drum and disc brakes, with disc brakes being the most common. In such a solution, the disc to which the road wheel is attached performs a rotational movement, and its angular velocity is equal to the velocity of the road wheel [17,18,19].

In order to maximize the effectiveness of the brakes, numerous tests are carried out with different materials to find the optimal combination of the friction pair. While the brake disc is simple, as it is usually grey cast iron, there is an unlimited number of variations with brake pads. It is a composite material, which usually consists of about 20 different components [20]. The components used can be divided according to their function into the following [21,22,23,24]:

- reinforcement—which is usually fibrous materials of natural or synthetic origin; currently, aramid or carbon fibers are most often used in this role,
- matrix—the binder of the whole, most often resin,
- fillers—fine materials that fill empty spaces between the other components, often fly ash fractions are used,
- and friction modifiers—determining the final tribological properties of the friction material.

The correct selection of friction modifiers is extremely important. Steel or cast iron are often used as additives, which are hard materials and cause an increase in the friction coefficient. Unfortunately, their presence causes adhesion on the friction surfaces, which leads to the brutal tearing out of material fragments from both the brake disc and the brake pad. The presence of steel and cast iron improves braking efficiency, but unfortunately accelerates the abrasive wear process [25,26,27].

To prevent this, components that reduce the friction coefficient are added to the pad composition. The most popular of them is copper. It plays two basic roles in the pad: (1) it dissipates heat from the contact zone, and (2) it leaves a thin layer on the contact surfaces, which prevents excessive adhesion and reduces unpleasant sounds—it is a kind of solid lubricant [28,29,30]. Unfortunately, although copper is an almost obligatory component of the pad, it is undesirable in friction materials. Due to its harmful properties, mainly for the aquatic environment, but also for humans, in whom it can cause multi-organ diseases [31,32,33,34].

Due to the above-mentioned harmfulness, numerous legal restrictions are introduced, limiting the percentage of copper in vehicle friction materials. Being aware of the upcoming changes, many researchers are trying to find a substitute for this material. Gilardi et al. carried out tests of various types of graphite, which they used as a solid lubricant in brake pads [35]. They prepared six groups of samples—one without graphite, while the remaining 8% contained graphite in various forms. It turned out that graphite provides good lubricating properties, which reduces unwanted squeaking. It also contributes to improving thermal conductivity. Mahale et al. attempted to replace copper with stainless steel swarf (SSS) [36]. A number of samples were made with different concentrations of this friction modifier, and then tests were performed to determine the physical, mechanical, and tribological properties. The tests showed that the proposed material has poorer resistance to fading. It is characterized by good anti-adhesive properties. The increase in SSS content also resulted in the improvement of tribological properties. Zheng et al. proposed a friction material in which copper was replaced by using the properties of fly ash cenospheres [37]. It turned out that the new component improves thermal stability, hardness and impact strength, reduced density of the final product, effectively increased the friction coefficient at medium and high temperatures, and enhanced the heat-fade resistance of the braking materials. It also provides a thin lubricating film on the cooperating surfaces. Chaurasiya et al. decided to check how aluminum would perform in the role of copper [38]. Four groups of samples with 0–15% aluminum content were prepared. Both physical properties (mechanical strength, hardness) and tribological properties were tested using the pin-on-disc association. It was shown that samples built on the basis of aluminum have lower density, friction coefficient, almost equal hardness, tensile strength, flexural strength and compression strength, and better stability with respect to friction coefficient. Braking materials based on aluminum were also studied by Strojny-Nędza et al. [39]. Four types of materials with different percentages of ceramic fraction were developed. However, the research focused primarily on the porosity and hardness of the material.

Analyzing the above literature reports, it was decided to compose proposals for friction materials in which copper was reduced and then completely eliminated from the composition. Its lubricating properties were replaced by using Teflon, and thermal conductivity was decided to be ensured by using aluminum.

The choice of aluminum was not accidental. Its excellent properties make it a very promising component that can be used in friction materials. A definite advantage is its low density. This is a desirable parameter in industry, as it has a direct impact on inertia, and therefore translates into vehicle acceleration and deceleration. Its high plasticity also translates into an advantage. Its Young’s modulus is about 40% smaller compared to copper. This contributes to increasing the surface of the pad–disc cooperation, reducing the number of roughness peaks. Unfortunately, aluminum is characterized by a slightly higher (by about 30%) thermal expansion, which in the case of reaching significant temperatures can negatively affect the integrity of the material. Aluminum is also characterized by excellent anti-corrosion properties, which is not without significance in the case of the working conditions of the braking system. Another advantage of aluminum is its high, although lower compared to copper, thermal conductivity. This property will provide the required cooling, especially in cases of sudden braking.

Teflon, in turn, is a material that provides excellent lubricating properties. Thanks to this, in the case of friction contact, vibrations are significantly reduced. It is also characterized by high chemical inertness. It is resistant to almost all chemical factors and solvents. It also does not show changes under the influence of UV radiation, which is important in the delicacy of vehicle brakes. It is also resistant to high temperatures, in the range of 250–300 °C. Most importantly, it is non-toxic, odorless, and tasteless. Therefore, it does not threaten the natural environment.

The aim of this work is to check whether the material prepared in this way will differ in tribological properties from materials based on copper, and if so, to what extent.

## 2. Materials and Methods

The subjects of the research described in this paper are proposals of prototype friction materials dedicated to braking systems. A total of four types of samples (S1..S4) were made, with three samples of each type. The composition of the individual samples was developed with the idea of replacing copper in mind. As already mentioned, copper plays two basic roles: solid lubricant and thermal conductor. Thanks to this, it is very difficult to replace. It was decided to check whether these functions can be taken over by other materials.

There are many substances with excellent lubricating properties. One of them is polytetrafluoroethylene (Teflon). It is a polymer with the chemical formula CF2CF2Jn. This substance has good anti-adhesive properties, which, together with good resistance to high temperatures, makes it a promising material. Powder (produced by Elube, Poland) with a grain size of 20–70 µm was used to produce the samples. According to the manufacturer, it withstands temperatures above 300 degrees. Its bulk density is 0.56 g/cm^3^. Its purity above 99.9%, and humidity is below 0.03%.

There are also many excellent thermal conductors available. A generally available material that meets this criterion is aluminum. It conducts thermal energy very well, and at the same time has a lower density. It also has excellent corrosion resistance. It is much more environmentally friendly than copper. A powder with a fineness of about 65 µm was used (produced by SYNTHETIKA SP.Z.O.O, Łódź, Poland).

For comparative purposes, a sample with a conventional composition was also made based on a copper friction modifier. The detailed composition of the samples made is presented in Table 1.

**Figure 1:**
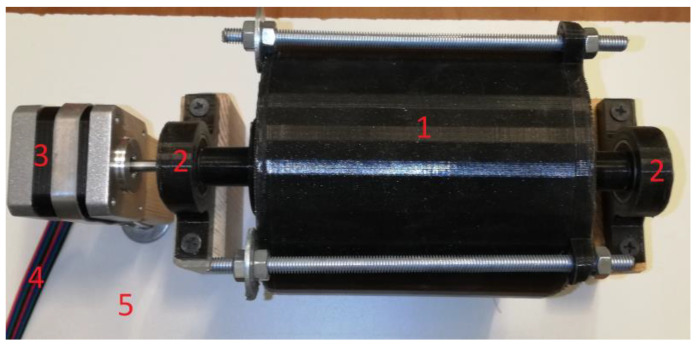
Component mixing device: 1—mixing tube, 2—bearing, 3—stepper motor, 4—connector, and 5—base.

As can be seen, sample S1 is characterized by a composition resembling commercial brake pads. It contains 20% copper powder. In samples S2..S4, copper was removed from the composition and replaced with aluminum powder and polytetrafluoroethylene powder in different proportions. This will allow us to determine how the proportions of both substances will affect the tribological properties of the final material. In each case, the appropriate amounts of materials were measured using a Steinberg SBS-LW-300A precision balance (accuracy 10^−3^ g, Hamburg, Germany). The measured material was placed in an additively manufactured mixing device (Figure 1). The internal ribs, at a rotational speed of 50RPM, thoroughly mixed the composite for one hour.

**Figure 2:**
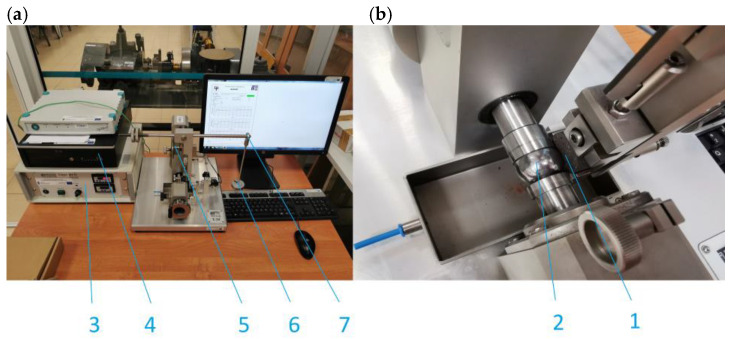
T-20 test stand: (**a**)—main components; (**b**)—friction pair; 1—sample; 2—counter sample; 3—control unit; 4—PC; 5—motor; 6—load; and 7—lever with counterweight.

The prepared material was placed in steel cylindrical molds with an internal diameter of 1” (25.4 mm). Then, a pressure of 20 MPa was exerted on the raw sample. The prepared samples were heated at a temperature of 55–60 °C for 24 h. The prepared samples were subjected to grinding of the front surfaces to obtain the required flatness tolerance and surface roughness at the level of 10^−6^ m. The T-20 laboratory stand implementing the ball-on-disc association was used for the tests (Figure 2). This stand has been used many times for this type of test, and the detailed methodology is described in [40].

In this method, proper preparation of the experiment is extremely important. Various methods are used here, but the most popular one is the experimental optimization of multi-parameter processes: the Taguchi method [41,42]. It allows to select such input parameters of the study for which the relative error will be the smallest. The first step is to prepare an orthogonal table. In this case, three input values are necessary: ball rotational speed (*n*), friction path (*S*), and pressure force (*L*). With three variables, the orthogonal table takes on nine rows [43]. The proposed table necessary for performing preliminary tests, developed on the basis of the standard [44], is presented in Table 2.

The preliminary tests were performed using a base sample in accordance with the above table, while the parameters were set in accordance with the above table. Each test was performed five times. The results obtained in the tests are presented in Table 3.

This was made possible by using the criterion “the bigger the better” described by the formula:

(1) $$\eta =-10{log}_{10}\left(\frac{1}{m}\sum_{i=1}^{m}x_{i}^{2}\right)$$

where *η* (ETA)—signal-to-noise ratio (S/N) function; *m*—number of measurements for a single sample; and *x*—average friction force value of a single test allowed for the designation of functions to determine the values of the ETA function.

**Figure 3:**
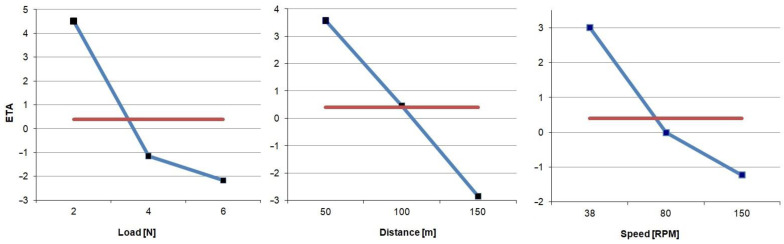
ETA function diagram: blue line—calculated value, red line—average.

The obtained results are presented in Figure 3. They allowed us to finally determine the input parameters of the proper experiment. They are finally as follows:

- load: 2 N,
- friction path: 50 m,
- ball rotation speed: 38 RPM.

**Figure 4:**
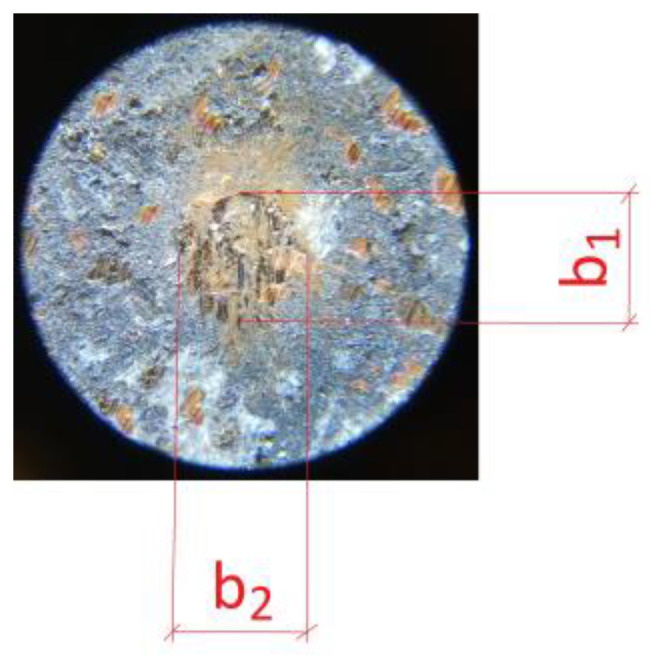
Measuring procedure on crater produced in ball-cratering method.

The next step was to determine the abrasive wear rate coefficient of the prepared materials. This is an extremely isotonic parameter, as it determines the service life of the brake pad. The sizes of the craters formed in the samples were used to determine it. The craters were measured in two planes: in line with the friction direction (*b*_1_) and perpendicular to it (*b*_2_). A Delta Optical microscope and a Brinell magnifying glass were used for this purpose (measuring example on Figure 4).

Finally, the crater size was determined as the arithmetic mean of both measurements:

(2) $$b=\frac{b_{1}+b_{2}}{2}$$

The determined value was substituted into the Archard equation, which after transformation takes the following form [44,45]:

(3) $$k_{c}=\pi \frac{b^{4}}{64RSL}$$

where *R*—the counter-sample radius (in this case ½ inch).

## 3. Results and Discussion

The direct results of tribological tests are time profiles of friction force values. Using the Coulomb and Moraine friction law with the following wording:

(4) $$f=\frac{\bar{F}}{N}$$

where$\;\bar{F}$—average friction force, and *N*—load, it was possible to calculate friction coefficient values in individual tests.

**Figure 5:**
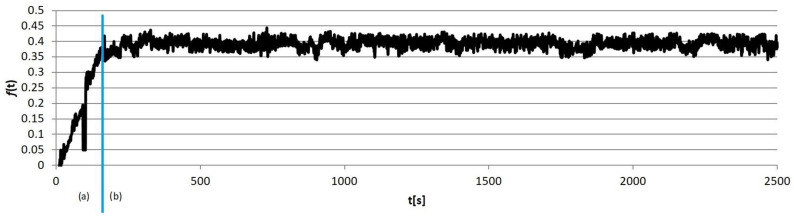
An example results graph of the coefficient of the friction of the sample from group S1: (a) running-in period, (b) proper friction.

**Figure 6:**
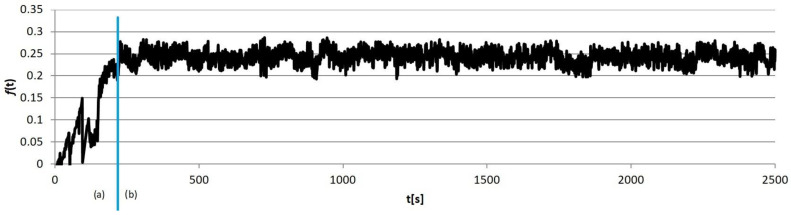
An example results graph of the coefficient of the friction of the sample from group S2: (a) running-in period, (b) proper friction.

**Figure 7:**
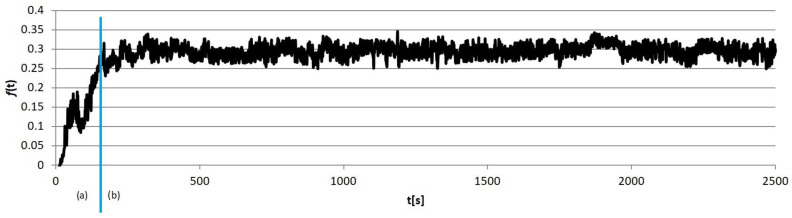
An example results graph of the coefficient of the friction of the sample from group S3: (a) running-in period, (b) proper friction.

**Figure 8:**
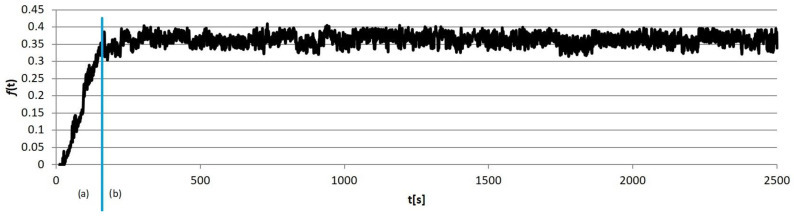
An example results graph of the coefficient of the friction of the sample from group S4: (a) running-in period, (b) proper friction.

Example calculation results obtained for individual sample groups are presented in Figure 5, Figure 6, Figure 7 and Figure 8. Each of them marked the running-in period (*a*), in which the geometrical “matching” of the sample and counter-sample took place. The clear end of this process resulted in the transition to the so-called proper friction. In further considerations, the running-in periods were rejected, and only the periods of proper friction were subjected to analysis.

Detailed results of the calculations of the average friction coefficient value for individual tests are presented in Table 4. For each group of samples, the average values were also determined, and then the standard deviation was determined using the following formula:

(5) $$S_{d}=\sqrt{\frac{\sum_{i=1}^{3}{\left(f_{j}-{\bar{f}}_{j}\right)}^{2}}{2}}$$

where *f_j_*—average COF value of a single sample.

Results are compiled in Table 4.

The above results show that the proposed change in the composition of the composite friction material affects the friction coefficient values. Replacing copper with a mixture of Teflon and aluminum in various proportions causes a decrease in the COF value. The largest decrease, by about 40%, was recorded at a Teflon concentration of 16% and a 4% concentration of aluminum powder. These data show that polytetrafluoroethylene provides the composite material with much better lubricating properties than copper. The decrease in the COF value in the proposed S2 material is much greater than, for example, when doping with potassium titanate [46]. Moghadam et al. obtained a decrease of about 20–30% (depending on the test conditions) in relation to the initial friction material containing copper. As it turns out, the Teflon doping is crucial here. Bhatt et al. [47] also used aluminum, but in combination with steel. They obtained slightly different results, namely the increase in the content of the alloy they proposed caused an increase in the friction coefficient.

The change in the concentration of Teflon and aluminum, in favor of aluminum, caused an increase in COF. It turned out that at an aluminum concentration of 16% and 4% polytetrafluoroethylene content (sample S4), the average friction coefficient was 0.353. This is less than a 10% decrease in relation to the initial sample containing copper (S1). Very similar results were obtained by Saikrishnan et al. [48] and Antonyraj et al. [49]. In both of these works, an attempt was made to replace copper with graphite, and in both of them, a decrease in the friction coefficient value was recorded.

**Figure 9:**
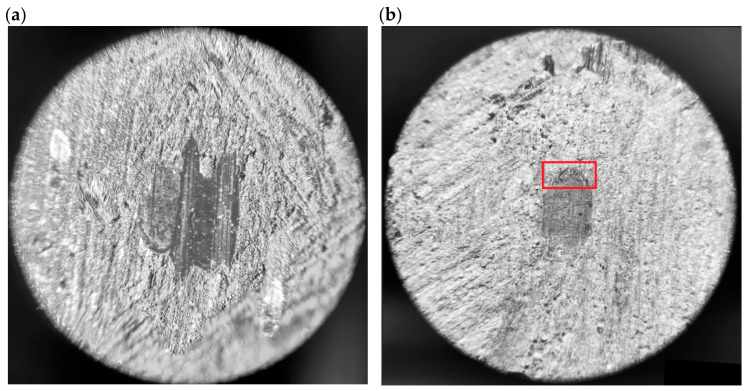

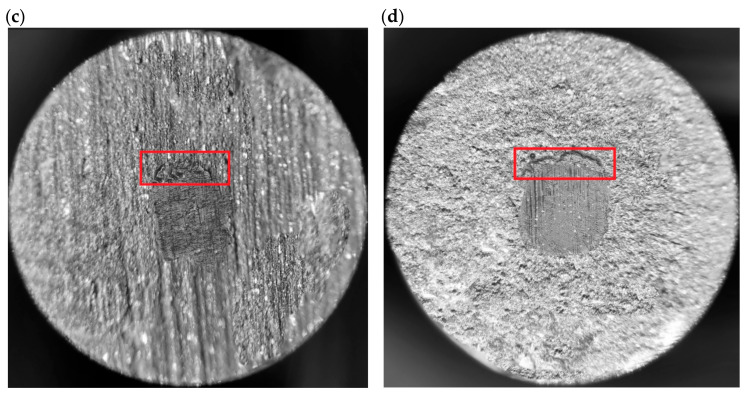
Craters on samples from group (**a**) S1, (**b**) S2, (**c**) S3, and (**d**) S4; the red line indicates the accumulation of lubricant material (40 × magnification).

Figure 9 shows photos of craters formed as a result of testing samples from individual groups. Clear differences can be seen in both their structure and shape. The sample containing copper (S1) was characterized by an irregular crater shape.

In samples containing Teflon grease and aluminum powder (S2..S4), the shape of the craters is more uniform, similar to a circle. In addition, a clear layering of material was noticeable on the extension of the friction trace. It was a mixture of wear products (components of the friction material) and Teflon grease. This mixture was deposited in the friction node, which reduced the coefficient of friction of the rubbing pair. This is a sign of better lubrication, but may suggest poorer structural integrity. Material tests planned at a later stage will allow for more precise verification of the material characteristics.

The friction tests were carried out at a constant temperature (approx. 21 °C) and constant humidity (approx. 40%). Although such a microclimate does not reflect the actual operating conditions of the braking system, our research was mainly aimed at a comparative aspect, which was achieved. We wanted to obtain similar measurement conditions for all groups of samples, which allowed us to eliminate the influence of the environment on the obtained results. In order to obtain a full picture of the suitability of the proposed material for brakes, it is necessary to perform tests at elevated temperatures, which is planned in the next stages.

In order to estimate the size of the influence of the type of component used, a statistical analysis was performed. Since the influence of the factor (type of lubricant and thermal conductor) on the selected parameter (COF—coefficient of friction) was studied, the most advantageous was the use of one-factor analysis of variance (ANOVA) [50,51]. It was assumed that the calculations would be performed with a degree of confidence of α = 95%. The first step was to determine the degrees of freedom for the qualitative factor, random terror, and total variation. The following dependencies were used for this purpose:

(6) $$D_{fa}=a-1$$

(7) $$D_{fe}=N-a$$

(8) $$D_{ft}=N-1$$

where *a*—the number of objects in the entire experiment, *N*—the number of experimental units in the entire experiment. The verification of the correctness of the calculation is the fulfillment of the equality:

(9) $$D_{fa}+D_{fe}=D_{ft}.$$

The above equation is true in the case studied, so it was possible to calculate the sums of squares. These calculations were also performed for three factors: the qualitative factor, random terror, and total variation. The following equations were used:

(10) $${SS}_{a}=\sum_{i=1}^{a}n\left({\bar{x}}_{i}-\bar{x}\right)$$

(11) $${SS}_{e}=\sum_{i=1}^{a}\sum_{j=1}^{n_{i}}\left(x_{ij}-{\bar{x}}_{i}\;\right)$$

(12) $${SS}_{t}=\sum_{i=1}^{a}\sum_{j=1}^{n_{i}}\left(x_{ij}-\bar{x}\;\right)$$

where *n*—number of repetitions, ${\bar{x}}_{i}$—object mean, $\bar{x}$—overall mean, and *x*—value of a single measurement for sample, and there must be a relation between *SS* values:

(13) $${SS}_{a}+{SS}_{e}={SS}_{t}.$$

Average values of qualitative factor and for random error took form:

(14) $${MS}_{a}={SS}_{a}/D_{fa}$$

(15) $${MS}_{e}={SS}_{e}/D_{fe}.$$

Results are summarized Table 5.

In the above table, it can be seen that the *p*-value is less than 0.05. This allows us to state that with the assumed confidence level of α = 95%, the hypothesis about the influence of the type of component used on the friction coefficient values should be accepted:

(16) $$H:\;f_{S1}\neq f_{S2}\neq f_{S3}\neq f_{S4}$$

Therefore, statistically significant differences were demonstrated between the friction coefficient values determined for the individual sample groups. Then, the homogeneity of the samples in each group was determined. For this purpose, the Levene test was used [51].

The results (Table 6) show that for all sample groups (S1..S4), the *p*-value assumes values that allow for unambiguous determination of the homogeneity of the results in the individual groups. This means that the differences in the *f* values are influenced only by the composition of the composite friction material.

In the next stage, the abrasive wear rate coefficient was calculated for individual groups of samples. For this purpose, it was necessary to measure the size of craters formed as a result of the test. By substituting the measurement results into Equations (2) and (3), the values of the *k_c_* coefficients for individual samples were obtained. Next, the mean values and standard deviations were calculated using the same methods as for the friction coefficients. The results are presented in Table 7.

**Figure 10:**
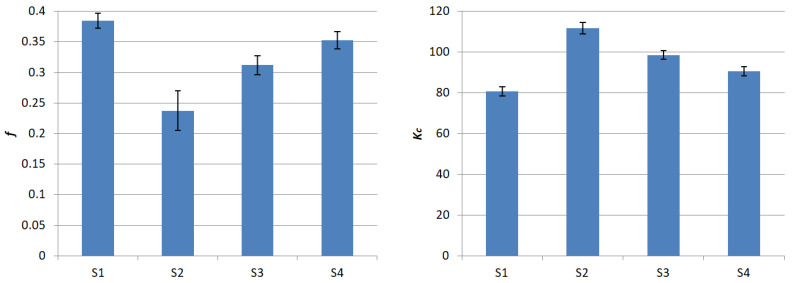
Summarized results of the coefficient of friction and abrasive wear rate.

The abrasive wear rate coefficient is a very important parameter. It determines the service life of the friction material, and therefore the frequency of replacement. In material S1, which was a kind of reference point, the value of the *k_c_* coefficient was 80.655 m^4^·m^−^^2^·N^−^^1^. Removing copper and replacing it with aluminum and Teflon grease resulted in an increase in the value of this coefficient. The smallest increase, by about 15%, was noted for material S4, where the Teflon concentration was the lowest. The largest, by as much as about 25%, was noted for the sample with the highest concentration of polytetrafluoroethylene. This fact results from the very low ability of Teflon to create adhesive bonds. It does not create permanent connections with other components, which reduces the internal integrity of the material. This feature is clearly visible in the above study. Unfortunately, this will have a negative impact on the service life of the working elements of the brake systems. It can be expected that it will be shortened by about 25%. However, this problem can be reduced, for example, by modifying the thickness of the friction pad. Although more material will be used in the production process, the service intervals will not change. It is worth adding that the obtained values are significantly higher than for the material using natural reinforcement in the form of flax fibers, which were proposed in previously published studies [52]. The obtained results are presented in Figure 10.

The problem with the materials proposed in this paper may be the cost. While aluminum is about 40% cheaper, unfortunately, Teflon is much more expensive than copper. On the market, the price of polytetrafluoroethylene is about twenty times higher than copper. Despite the fact that there is not much of it in the friction material, the difference in the price of the final product may be significant. Despite this difference, when the ban on the use of copper is introduced, the use of a mixture of aluminum and Teflon may prove to be an interesting and important alternative.

## 4. Conclusions

The paper proposes prototype friction materials. The main goal was to eliminate copper from the composition, which is a material harmful primarily to aquatic life, but also to humans. Its lubricating properties were replaced by using Teflon grease (polytetrafluoroethylene), while its thermal conductivity was replaced by using aluminum powder. Samples were made with different percentage concentrations of the above materials (S2…S4), and a sample with a conventional composition (S1) as a reference. Here, the copper content was 20%. Laboratory tests were performed using the ball-cratering contact methodology. It allows for determining such tribological features as the coefficient of friction and the abrasive wear rate coefficient. It was found that:

- samples with a conventional composition, containing 20% copper (S1), were characterized by the highest coefficient of friction, amounting to 0.384,
- removing copper and replacing it with Teflon grease and aluminum caused a decrease in the COF value; the lowest value was recorded for the composition containing 4% aluminum and 16% Teflon grease (sample group S2); the friction coefficient value was then 0.237,
- the friction coefficient value closest to the starting material, i.e., 0.353, was obtained for samples from group S4, where 16% polytetrafluoroethylene and 4% aluminum were used,
- doping the samples with alternative components resulted in an increase in the abrasive wear rate coefficient in each case,
- and the highest *k_c_* coefficient value was obtained for material S2, while the lowest was obtained for S4.

The next stage of the research will include temperature tests, in which it is planned to determine the temperature profiles of friction heating in the braking process using the proposed materials. Depending on the results obtained, composition optimization or strength tests will be proposed. Their aim will be to check the mechanical properties of prototype composite friction materials.

## Figures and Tables

**Table 1:** Compositions of samples S1..S4.

Component	S1, wt%	S2, wt%	S3, wt%	S4, wt%
Cooper powder, grain < 60 µm (Cu)	20	0	0	0
Aluminum powder, grain ~65 µm (Al)	0	4	10	16
Polytetrafluoroethylene powder, grain 20–70 µm (CF2CF2Jn)	0	16	10	4
Steel chips, size 0.5–1 mm (0.18% C, 0.5% Si, 1.65% Mn, 0.05% P, 0.02% S, 0.08% Mo)	11	11	11	11
Aramid fiber, length ~5 mm	18	18	18	18
Resin	19	19	19	19
Fly ash, grain 1–200 µm	23	23	23	23
Cast iron powder, grain < 200 µm (EN-GJS-400-12)	9	9	9	9

**Table 2:** Orthogonal table for preliminary tests.

Test No.	*L*, N	*S*, m	*N*, RPM
1	2	50	38
2	2	100	80
3	2	150	150
4	4	50	80
5	4	100	150
6	4	150	38
7	6	50	38
8	6	100	150
9	6	150	80

**Table 3:** Preliminary tests results.

Test No.	Average Crater Diameter [mm] at Repeat No.				
	1	2	3	4	5
1	0.39	0.37	0.40	0.41	0.38
2	0.53	0.56	0.58	0.53	0.58
3	1.06	1.09	1.18	1.18	1.15
4	0.91	1.26	1.19	1.17	1.22
5	1.41	1.45	1.31	1.37	1.31
6	1.28	1.11	1.35	1.45	1.33
7	0.86	1.28	1.14	1.38	1.06
8	0.85	0.86	0.84	0.71	0.82
9	1.93	1.85	1.88	1.86	1.91

**Table 4:** Average *f* in individual tests.

Group No	Sample No	Coefficient of Friction					Average	Standard Deviation
		Run No 1	Run No 2	Run No 3	Run No 4	Run No 5		
S1	1	0.38	0.39	0.39	0.37	0.41	0.384	±0.0124
	2	0.39	0.37	0.38	0.39	0.4		
	3	0.37	0.38	0.4	0.38	0.37		
S2	1	0.23	0.19	0.23	0.28	0.21	0.237	±0.0324
	2	0.26	0.26	0.22	0.23	0.26		
	3	0.25	0.21	0.31	0.22	0.2		
S3	1	0.31	0.29	0.29	0.34	0.32	0.312	±0.0157
	2	0.3	0.32	0.29	0.31	0.33		
	3	0.32	0.33	0.3	0.31	0.32		
S4	1	0.36	0.34	0.36	0.36	0.37	0.353	±0.0144
	2	0.32	0.34	0.37	0.37	0.34		
	3	0.34	0.36	0.35	0.35	0.36		

**Table 5:** Single dimension ANOVA analysis calculations results.

Source of Variation	*Df*	*SS*	*MS*	*F_f_*	*p*
qualitative factor	3	18.35 × 10^−2^	61.18 × 10^−3^	141.57	0
random error	56	24.20 × 10^−3^	0.43 × 10^−4^		
total	59	20.77 × 10^−2^			

**Table 6:** Levene test results.

	Samples Group			
	S1	S2	S3	S4
*p*	0.382	0.285	0.308	0.365

**Table 7:** Calculated wear coefficients values.

Group No	Sample No	*k_c_* [m^4^·m^−2^·N^−1^]					Average	Standard Deviation
		Run No 1	Run No 2	Run No 3	Run No 4	Run No 5		
S1	1	81.58	79.24	76.55	78.88	81.71	80.655	±2.224
	2	80.64	83.49	79.12	79.97	83.39		
	3	82.47	81.95	79.89	83.74	77.21		
S2	1	110.33	109.57	115.72	112.68	110.75	111.811	±2.790
	2	108.67	114.38	109.94	111.35	112.93		
	3	112.96	116.39	107.41	108.77	115.31		
S3	1	104.91	97.78	96.71	97.59	99.24	98.586	±2.098
	2	98.74	96.23	100.31	98.54	97.35		
	3	99.36	97.32	96.78	99.28	98.65		
S4	1	87.39	87.67	92.76	90.81	91.44	90,579	±2.179
	2	89.66	93.47	91.55	88.62	90.68		
	3	91.24	92.11	94.39	87.48	89.41		

## Data Availability

The original contributions presented in the study are included in the article, further inquiries can be directed to the corresponding author.
